# Methylprednisolone Stiffens Aortas in Lipopolysaccharide-Induced Chronic Inflammation in Rats

**DOI:** 10.1371/journal.pone.0069636

**Published:** 2013-07-17

**Authors:** Ya-Hui Ko, Ming-Shian Tsai, Po-Huang Lee, Jin-Tung Liang, Kuo-Chu Chang

**Affiliations:** 1 Department of Physiology, College of Medicine, National Taiwan University, Taipei, Taiwan; 2 School of Chinese Medicine for Post-Baccalaureate I-Shou University and Department of Surgery, E-Da Hospital, Kaohsiung, Taiwan; 3 Department of Surgery, National Taiwan University Hospital, Taipei, Taiwan; University of Louisville, United States of America

## Abstract

**Introduction:**

Glucocorticoids are commonly used as therapeutic agents in many acute and chronic inflammatory and auto-immune diseases. The current study investigated the effects of methylprednisolone (a synthetic glucocorticoid) on aortic distensibility and vascular resistance in lipopolysaccharide-induced chronic inflammation in male Wistar rats.

**Methods:**

Chronic inflammation was induced by implanting a subcutaneous slow-release ALZET osmotic pump (1 mg kg^−1^ day^−1^ lipopolysaccharide) for either 2 or 4 weeks. Arterial wave transit time (τ) was derived to describe the elastic properties of aortas using the impulse response function of the filtered aortic input impedance spectra.

**Results:**

Long-term lipopolysaccharide challenge enhanced the expression of advanced glycation end products (AGEs) in the aortas. Lipopolysaccharide also upregulated the inducible form of nitric oxide synthase to produce high levels of nitric oxide (NO), which resulted in vasodilation, as evidenced by the fall in total peripheral resistance (*R_p_*). However, lipopolysaccharide challenge did not influence the elastic properties of aortas, as shown by the unaltered τ. The NO-mediated vascular relaxation may counterbalance the AGEs-induced arterial stiffening so that the aortic distensibility remained unaltered. Treating lipopolysaccharide-challenged rats with methylprednisolone prevented peripheral vasodilation because of its ability to increase *R_p_*. However, methylprednisolone produced an increase in aorta stiffness, as manifested by the significant decline in τ. The diminished aortic distensibility by methylprednisolone paralleled a significant reduction in NO plasma levels, in the absence of any significant changes in AGEs content.

**Conclusion:**

Methylprednisolone stiffens aortas and elastic arteries in lipopolysaccharide-induced chronic inflammation in rats, for NO activity may be dominant as a counteraction of AGEs.

## Introduction

An *in vivo* animal model of systemic inflammation in which lipopolysaccharide (LPS) is infused is useful for studying the integrative mediator pathways of inflammation as well as hemodynamic and functional changes in acute and chronic inflammatory disorders. LPS is the major bioactive component of the cell surface of gram-negative bacteria and is known to play a pivotal role in initiating a variety of host responses [1]. When LPS binding protein binds LPS to its receptors, especially Toll-like receptor (TLR)-4, downstream intracellular signaling pathways are initiated; the ultimate result is the activation of nuclear factor-κB (NF-κB) [2].

The translocation of NF-κB to the nucleus results in an upregulation of pro-inflammatory cytokines, including tumor necrosis factor (TNF)-α, C-reactive protein (CRP), and interleukin (IL)-6 [3]. Meanwhile, NF-κB activation enhances the expression of receptor for advanced glycation end products (RAGE) as NF-κB possesses a binding site for the *RAGE* gene [4]. Binding of RAGE by its ligands such as advanced glycation end products (AGEs) produces reactive oxygen species, which further activates NF-κB to amplify RAGE signal transduction. Moreover, the increased oxidant stress accelerates AGEs formation, which can modify matrix proteins to encourage the retention of inflammatory cells in the vessel wall [5]. Thus, the RAGE-AGE interaction associated with LPS stimulation may maintain and even amplify inflammatory activities, critically leading to vascular dysfunction.

Many cell types express inducible form of nitric oxide synthase (iNOS) when exposed to bacterial products or pro-inflammatory cytokines [6]. In inflammation, high and prolonged production of nitric oxide (NO) may lead to cytotoxic and pro-inflammatory effects. In vascular rings, rats treated with LPS showed a marked induction of iNOS [7]. High levels of NO produced by iNOS may exert a detrimental effect on the contractile status of vascular smooth muscle cells (VSMCs). Moreover, NO may react with superoxide to generate highly toxic compounds such as peroxynitrite to damage arterial trees [8]. Although AGEs has the ability to quench NO, the exact mechanism by which their interaction lead to hemodynamic changes under LPS has not been fully explored in intact animals.

Glucocorticoids are commonly used as therapeutic agents in many acute and chronic inflammatory and auto-immune diseases [9]. Their therapeutic action has largely been attributed to their anti-inflammatory and immunosuppressive efficacy. Glucocorticoids inhibit the production of inflammatory cytokines induced by LPS-activated monocytes/macrophages and protect animals from LPS-induced lethality [10]. Methylprednisolone (MP) is a synthetic glucocorticoid and is a powerful anti-inflammatory agent that inhibits NF-κB activation, thereby suppresses iNOS expression and other inflammatory factors [11]. However, whether MP effects on NO production and AGEs formation are involved in the beneficial MP action in improving vascular function remains to be determined.

AGEs are the products of nonenzymatic glycation and oxidation of proteins, which may form over a period of weeks [12]. Thus, the aim of this study was designed to determine the anti-inflammatory effects of MP on aortic distensibility and vascular resistance in LPS-induced chronic inflammation in rats. Chronic inflammation was induced by implanting a subcutaneous slow-release ALZET osmotic pump for either 2 or 4 weeks. The physical properties of the arterial system were assessed by making use of the aortic input impedance spectrum that is the frequency relationship between pulsatile aortic pressure and flow signals [13], [14]. Arterial wave transit time was derived to describe the elastic properties of aortas and large arteries. NO plasma levels and AGEs content within the vessel wall were also detected.

## Materials and Methods

### 1. General Preparation

Animals. Male Wistar rats weighing 250 to 300 g were randomly divided into three categories as follows (*n* = 10 in each group): (i) sham groups, (ii) LPS groups, and (iii) LPS groups treated with MP. For the LPS groups, chronic inflammation was induced by implanting a subcutaneous slow-release ALZET mini osmotic pump (Model 2004; DURECT Corporation, Cupertino, CA) to infuse LPS (*E. coli* O55:B5, 1 mg kg^−1^ day^−1^; Sigma-aldrich, Missouri, USA) for either 2 or 4 weeks. Saline infusion was used in the sham groups. For the LPS-MP groups, rats received a daily injection of MP (5 mg kg^−1^ day^−1^, i.p.; Pfizer Manufacturing Belgium, NV) as anti-inflammatory therapy, injected into the abdominal cavity. Animals were allowed free access to Purina chow and water with a 12-h light/dark cycle. The experiments were conducted according to the *Guide for the Care and Use of Laboratory Animals*, and our study protocol was approved by the Animal Care and Use Committee of National Taiwan University.

Enzyme-linked immunosorbent assay (ELISA) for plasma CRP, IL-6, NO and Peroxynitrite. Quantification of plasma levels of CRP (ALPCO, NH), IL-6 (R&D Systems, MN, USA), NO (Nitrites+Nitrates) (Calbiochem, Merck, Germany) and peroxynitrite (Cayman Chemical, MI, USA) were performed using commercially available ELISA kits in strict accordance with the manufacturer’s instructions [15]–[18].

#### Immunofluorescence staining for iNOS

Rat aortic rings were fixed in 4% (w/v) formalin and embedded in paraffin. For immunoperoxidase labeling, after being washed with 1X PBS, the sections were treated with 3% H_2_O_2_ for 10 min to quench endogenous peroxidase; thereafter they were incubated in 2% normal horse serum (Santa Cruz Biotechnology, Inc., Santa Cruz, CA, USA) for 30 h to block nonspecific antigen binding. The sections were then reacted with primary polyclonal antibodies: anti-rabbit iNOS IgG (1∶100, Abcam, Cambridge, UK) in TBS containing 0.1% Triton X-100 for 1 h at room temperature. The sections were then incubated with the secondary antibodies for 30 min at room temperature by anti-rabbit conjugated rodamine. Sections were finally DAPI (1∶5000, Sigma, St. Louis, MO, USA) stained to label the nuclei. All tissue sections were mounted on gelatin-coated slides (Dako Cytomation) and embedded with Permount (Fisher; SP-15-100).

#### Immunohistochemical staining for AGEs and RAGE

Rat aortic rings were fixed in 4% (w/v) formalin and embedded in paraffin. The method for immunoperoxidase labeling has been described previously. The sections were then reacted overnight at 4°C with primary polyclonal antibodies, namely rabbit anti-AGE IgG (ab23722) (1∶10000; Abcam, Cambridge, UK) and goat anti-human RAGE IgG (1∶400; AbD Serotec., UK), all in TBS containing 0.1% Triton X-100. The sections were then incubated with secondary antibody Fab’ fragments for 1 h at room temperature using Histofine Simple Stain MAX PO (M) (anti- rabbit/mouse) (Nichirei Biosciences Inc., Tokyo, Japan) following the manufacturer’s instructions. Finally, sections were visualized with 3, 3′-diaminobenzidine tetrahydrochloride hydrate (DAB; Dako Cytomation; K3466). Hematoxylin nuclear staining (Sigma-aldrich, Missouri, USA) was also applied. All tissue sections were mounted on gelatin-coated slides (Dako Cytomation) and embedded with Permount (Fisher; SP-15-100).

#### Western blot analysis for iNOS, AGEs, and RAGE

Rat aortic tissues were pulverized at −80°C by using a pestle and mortar and resuspended in RIPA buffer (1% Nonidet P-40, 0.5% sodium deoxycholate, 0.1% SDS and 1% protease inhibitors cocktail). The homogenates were centrifuged at 12,000×g, 4°C for 15 min and supernatant was collected. The total protein concentration of the supernatant was determined by using the Bradford reagent (Sigma-aldrich, Missouri, USA). Proteins (50 µg each lane) were separated by 8% SDS-polyacrylamide gels electrophoresis (Mini-Protein III, Bio-Rad) and electrotransferred onto 0.2 µm PVDF membrane (Bio-Rad, Hercules, CA). The membranes were blocked overnight with 5% (w/v) nonfat milk in PBST buffer (PBS buffer with 0.05% (w/v) Tween 20) and incubated overnight with primary antibodies: rabbit polyclonal anti-AGEs antibody (ab23722) (1∶200; Abcam, Cambridge, UK), anti-RAGE antibody (ab37647) (1∶1000; Abcam, Cambridge, UK) or rabbit polyclonal anti-iNOS antibody (1∶100; Abcam, Cambridge, UK). The membranes were exposed to horseradish peroxidase-conjugated anti-rabbit IgG secondary antibody (1∶2000; Abcam, Cambridge, UK) for 1 hour, and immunoreactivity was visualized using an ECL detection system (PerkinElmer, MA, USA). Autoradiographic films were volume-integrated within a linear range of exposure using a scanning densitometer. Relative quantity was obtained by normalizing the density of target protein against that of β-actin. Experiments were replicated three times, and results were expressed as mean±s.e.

### 2. Catheterization

General surgical procedures and measurement of hemodynamic variables in anesthetized rats have previously been described [19]. Animals were anesthetized with intraperitoneal sodium pentobarbital (50 mg kg^−1^), placed on a heating pad, intubated, and ventilated with a rodent respirator (Model 131; New England Medical Instruments, Medway, MA, USA). The rectal temperature of each rat was monitored. The chest was opened through the second intercostal space of the right side. An electromagnetic flow probe (model 100 series, internal circumference 8 mm, Carolina Medical Electronics, King, NC) was positioned around the ascending aorta to measure the pulsatile aortic flow. A high-fidelity pressure catheter (model SPC 320, size 2F; Millar Instruments, Houston, TX, USA) was used to measure the pulsatile aortic pressure via the isolated carotid artery of the right side. The electrocardiogram (ECG) of lead II was recorded with a Gould ECG/Biotach amplifier (Gould Electronics, Cleveland, OH, USA). The selective pressure and flow signals from 5 to 10 beats were averaged in the time domain, using the peak R wave of ECG as a fiducial point. Timing asynchronicity between the pressure and flow signals (caused by the spatial distance between the flow probe and the proximal aortic pressure transducer) was corrected by a time-domain approach, in which the foot of the pressure waveform was realigned with that of the flow [20]. The resulting pressure and flow signals were subjected to further vascular impedance analysis.

At the end of the experiment, each rat was sacrificed to obtain the weight of left ventricle (LV). Ratio of the LV weight to body weight was used as an indicator for the degree of cardiac hypertrophy.

### 3. Aortic Input Impedance Spectra

**Figure 1:**
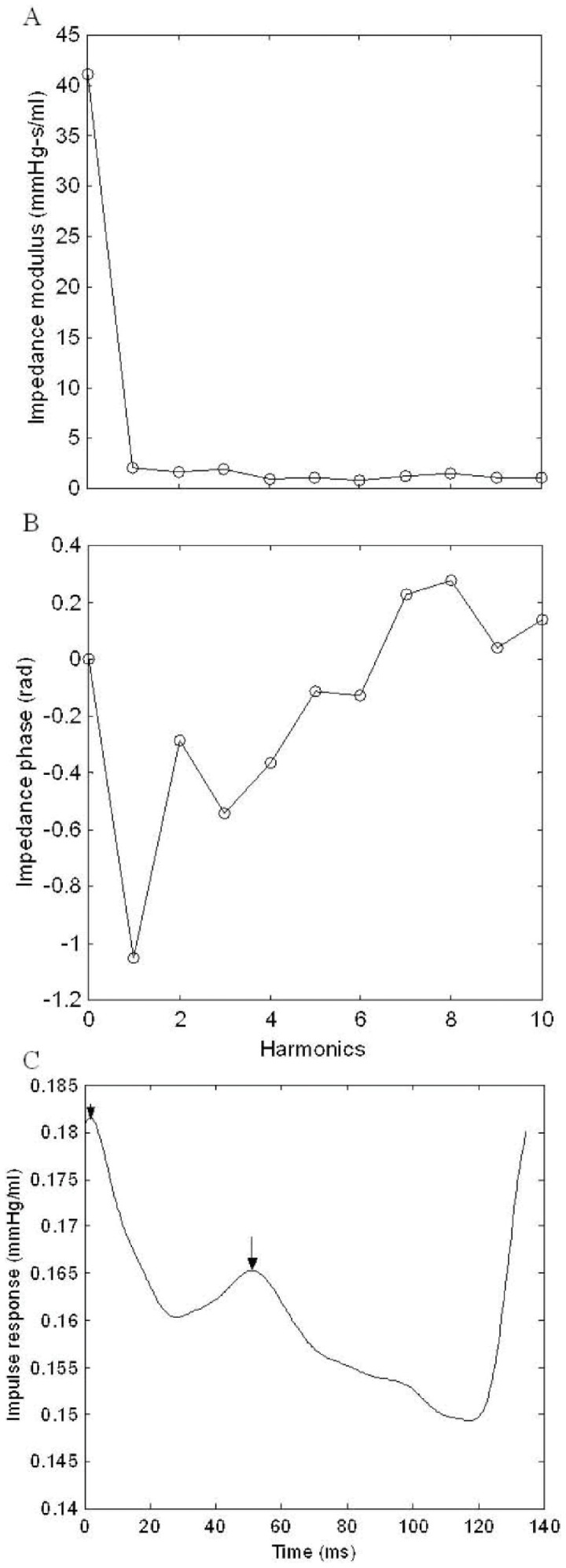
Modulus (A) and phase (B) of the aortic input impedance in a rat from the sham group, and impulse response function curve (C) derived from filtered aortic input impedance spectra shown in A and B. In C, the long arrow shows the discrete reflection peak from the body circulation and the short arrow demonstrates the initial peak as a reference. Half of the time difference between the appearance of the reflected peak and the initial peak approximates the arterial wave transit time (τ) in lower body circulation.



The aortic input impedance spectra (*Z_i_*) were obtained from the ratio of ascending aortic pressure harmonics to the corresponding flow harmonics (Fig. 1A and 1B) using a standard Fourier series expansion technique [13], [14], [21]. The total peripheral resistance of systemic circulation (*R_p_*) was calculated as the mean aortic pressure divided by the mean aortic flow. The aortic characteristic impedance (*Z_c_*) was computed by averaging high-frequency moduli of the aortic input impedance data points (4th−10th harmonics). Considering *Z_c_*, we calculated the systemic arterial compliance *C_m_* at mean aortic pressure *P_m_*, as follows:

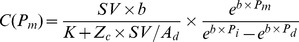
.

*SV* is the stroke volume; *K* is the ratio of the total area under the aortic pressure curve to the diastolic area (*A_d_*); *b* is the coefficient in the pressure-volume relation (−0.0131±0.009 in aortic arch); *P_i_* is the pressure at the time of incisura and *P_d_* is the end-diastolic pressure [19], [22].

The wave transit time (τ) was computed by the impulse response of the filtered *Z_i_* (Fig. 1C). This calculation was accomplished by the inverse transformation of *Z_i_* after multiplication of the first 12 harmonics by a Dolph-Chebychev weighting function with order 24 [23]. The long arrow shows the discrete reflection peak from the body circulation and the short arrow demonstrates the initial peak as a reference. Half of the time difference between the long and short arrows approximates the arterial τ in the lower body circulation [24]. The time domain reflection factor (*R_f_*) was derived from the amplitude ratio of backward-to-forward peak pressure waves using the method proposed by Westerhof *et al.* [25]. Therefore, both the wave transit time and the wave reflection factor characterized the wave reflection phenomenon in the vasculature.

### 4. Statistics

Results are expressed as mean±s.e. Analysis of variance (ANOVA) was used to determine the statistical significance while multiple comparisons were made among the effects of LPS and MP on hemodynamic and biochemical data. Significant differences were assumed at the level of *p*<0.05. If ANOVA results for any hemodynamic or biochemical variable reached this level of significance, then Tukey’s honestly significant difference method was used to determine which groups of rats have different mean values of that variable.

## Results

Table 1 shows the effects of LPS and MP on body weight (BW), LV weight (LVW) and rectal temperature (RT) in the rats studied. The LPS-challenged animals exhibited no significant difference in BW, LVW and LVW/BW ratio from that of the age-matched shams. Although MP therapy produced a marked decrease in BW and LVW, it did not alter LVW/BW in LPS rats. By contrast, treating LPS-challenged animals with MP for 4 weeks prevented an elevation in RT.

**Table 1:** Body weight (BW, g), left ventricular weight (LVW, g), ratio of the LVW to BW (LVW/BW, mg/g), and rectal temperature (RT, °C) in the animals.

Time point	2 weeks			4 weeks		
Group (*n* = 10)	Sham	LPS	LPS-MP	Sham	LPS	LPS-MP
**BW**	355.0±9.8	376.2±9.3	318.8±7.2‡	403.1±9.6	395.0±7.0	374.6±6.9‡
**LVW**	0.66±0.02	0.69±0.03	0.61±0.02‡	0.72±0.03	0.72±0.02	0.67±0.02
**LVW/BW**	1.85±0.05	1.89±0.07	1.91±0.06	1.80±0.05	1.77±0.05	1.87±0.04
**RT**	36.2±0.2	36.6±0.2	36.5±0.2	36.1±0.1	36.9±0.2†	36.3±0.3‡

All values are expressed as mean±s.e. LPS-MP, LPS rats treated with MP; LPS, lipopolysaccharide; MP, methylprednisolone.

† *p*<0.05 from the Sham group;

‡ *p*<0.05 from the LPS group.

Figure 1 exemplifies the *Z_i_* and the corresponding impulse response function curve for a rat from the sham group. The aortic impedance modulus fell steeply from a high value at zero frequency to extremely low values at frequencies that fluctuated around the *Z_c_* (Fig. 1A). The aortic impedance phase shown in Fig. 1B indicates the delay between the corresponding pressure and flow components. Figure 1C shows the impulse response function curve derived from the filtered *Z_i_*. Half of the time difference between the long and short arrows approximates the arterial τ in the lower body circulation.

**Figure 2:**
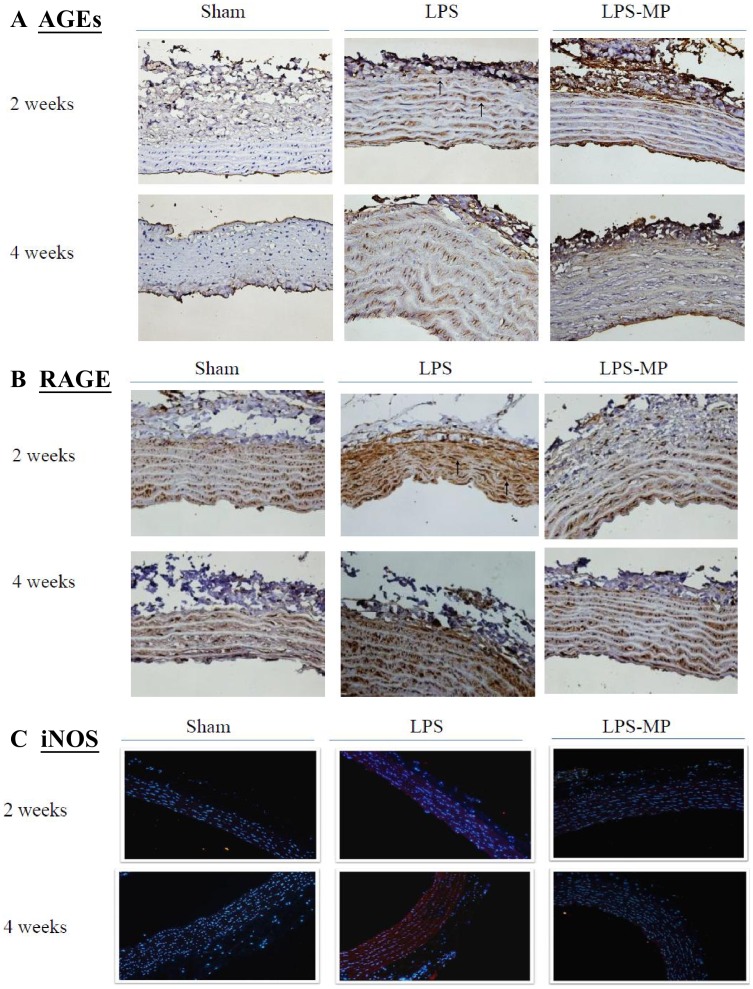
Effects of LPS and MP on the expression of AGEs (A), RAGE (B), and iNOS (C) in the aortas. AGEs and RAGE expressions were probed using immunohistochemical staining (400x). The arrows indicated the sites of antibody staining. iNOS expression (red color) was explored using immunocytochemical fluorescence staining (200x). LPS, lipopolysaccharide; MP, methylprednisolone; AGEs, advanced glycation end products; RAGE, receptor for AGEs; iNOS, inducible nitric oxide synthase.

**Figure 3:**
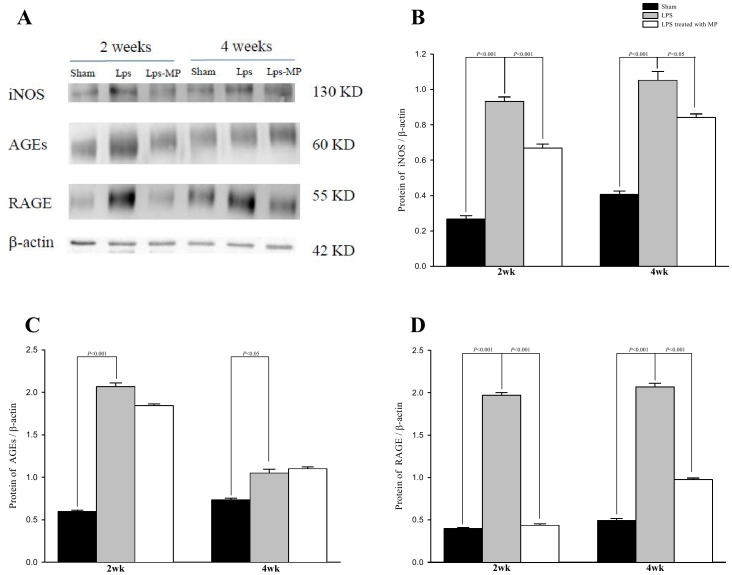
Effects of LPS and MP on aortic AGEs, RAGE, and iNOS proteins measured by Western blotting technique. Protein expression was normalized to β-actin. Data are expressed as mean±s.e. LPS, lipopolysaccharide; MP, methylprednisolone; LM, LPS groups treated with MP; iNOS, inducible nitric oxide synthase; AGEs, advanced glycation end products; RAGE, receptor for AGEs. (*n* = 10 in each group).

Figure 2 shows the effects of LPS and MP on the expression of AGEs (A), RAGE (B), and iNOS (C) in the aortas. The LPS-challenged rats displayed greater immunoreactivity of these proteins than did the aged-matched shams. The results were congruent with those obtained by Western blotting technique, which were shown in Fig. 3. Although MP prevented an LPS-related increase in RAGE and iNOS expression, it did not significantly attenuate the AGEs content in the LPS-challenged rats.

**Figure 4:**
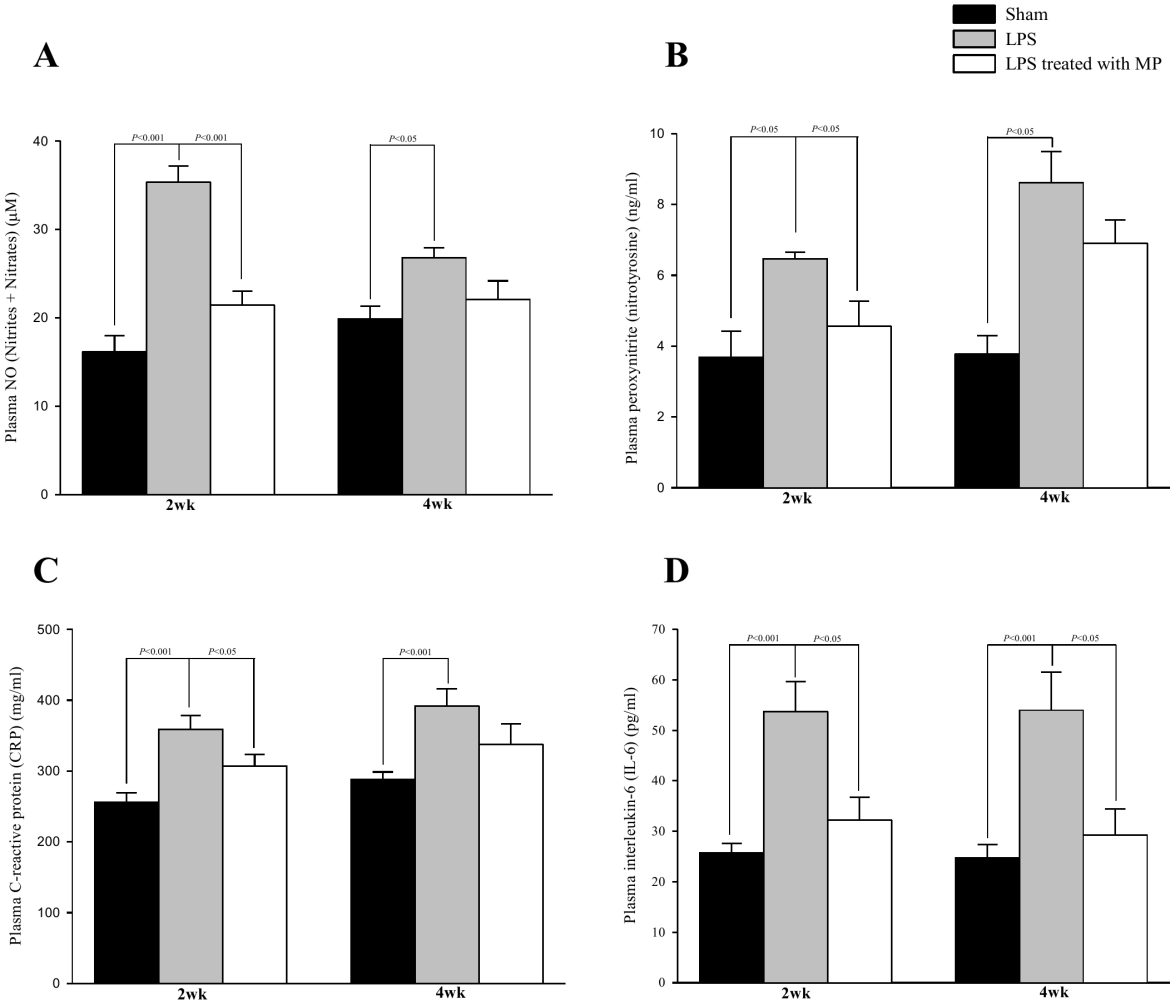
Effects of LPS and MP on the plasma levels of NO (Nitrites+Nitrates) (A), Peroxynitrite (nitrotyrosin) (B), CRP (C), and IL-6 (D). Data are expressed as mean±s.e. LPS, lipopolysaccharide; MP, methylprednisolone; NO, nitric oxide; CRP, C-reactive protein; IL-6, interleukin-6. (*n* = 10 in each group).

Figure 4 shows the effects of LPS and MP on the plasma levels of NO (A), peroxynitrite (B), CRP (C), and IL-6 (D). In rats challenged with LPS, the increase in NO and peroxynitrite was attenuated by treatment with MP. MP therapy also prevented the LPS-induced increase in those CRP and IL-6 cytokines in the plasma.

**Figure 5:**
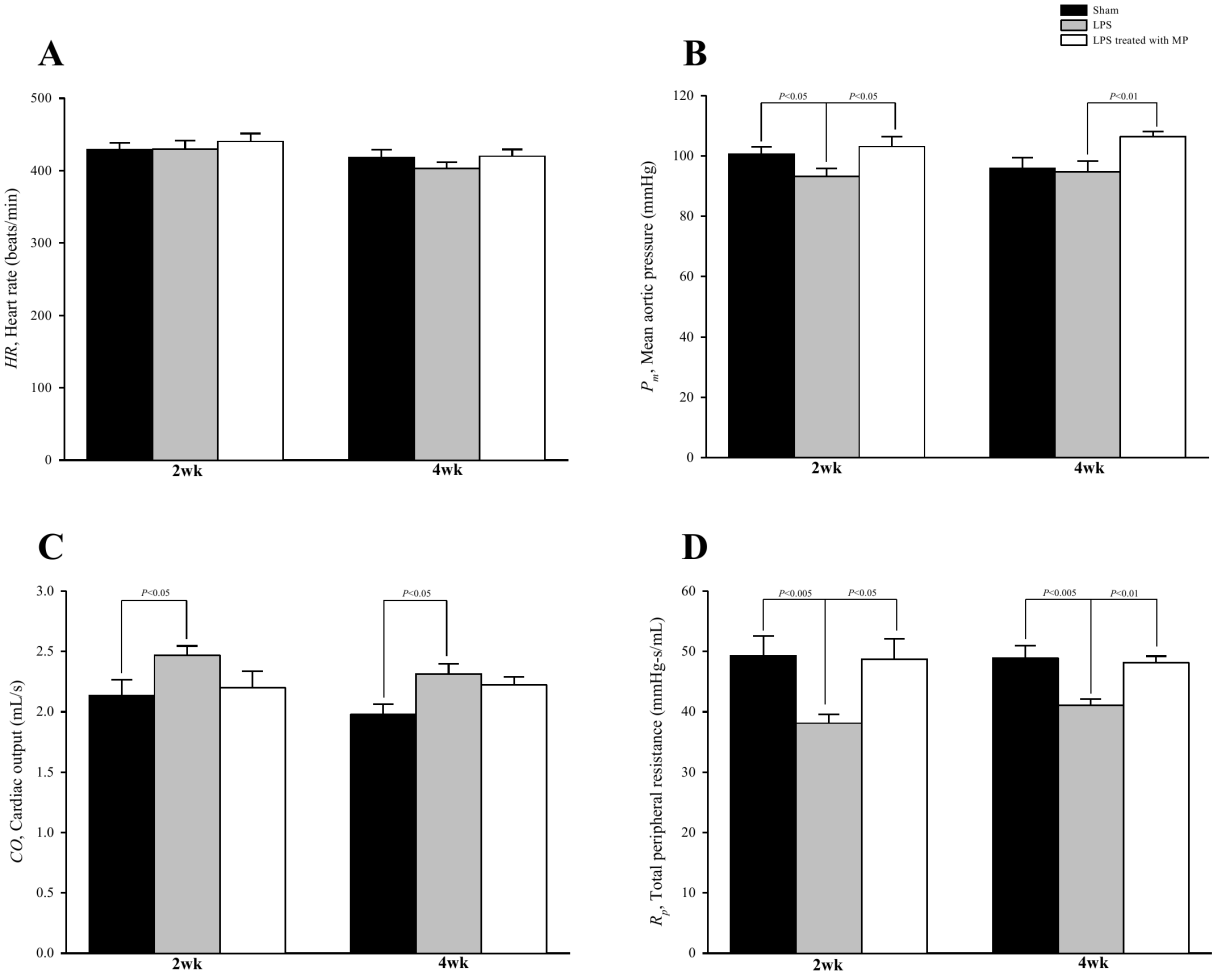
Effects of LPS and MP on basal heart rate (*HR* in A), mean aortic pressure (*P_m_* in B), cardiac output (*CO* in C) and total peripheral resistance (*R_p_* in D). Data are expressed as mean±s.e. LPS, lipopolysaccharide; MP, methylprednisolone. (*n* = 10 in each group).

Figure 5 shows the effects of LPS and MP on basal heart rate (*HR* in A), mean aortic pressure (*P_m_* in B), cardiac output (*CO* in C) and total peripheral resistance (*R_p_* in D). Neither LPS nor MP affected *HR*. Both the decreased *P_m_* and the increased *CO* contributed to a fall in *R_p_* in the LPS rats. MP therapy prevented LPS-induced peripheral vasodilation, as evidenced by the increased *R_p_*.

**Figure 6:**
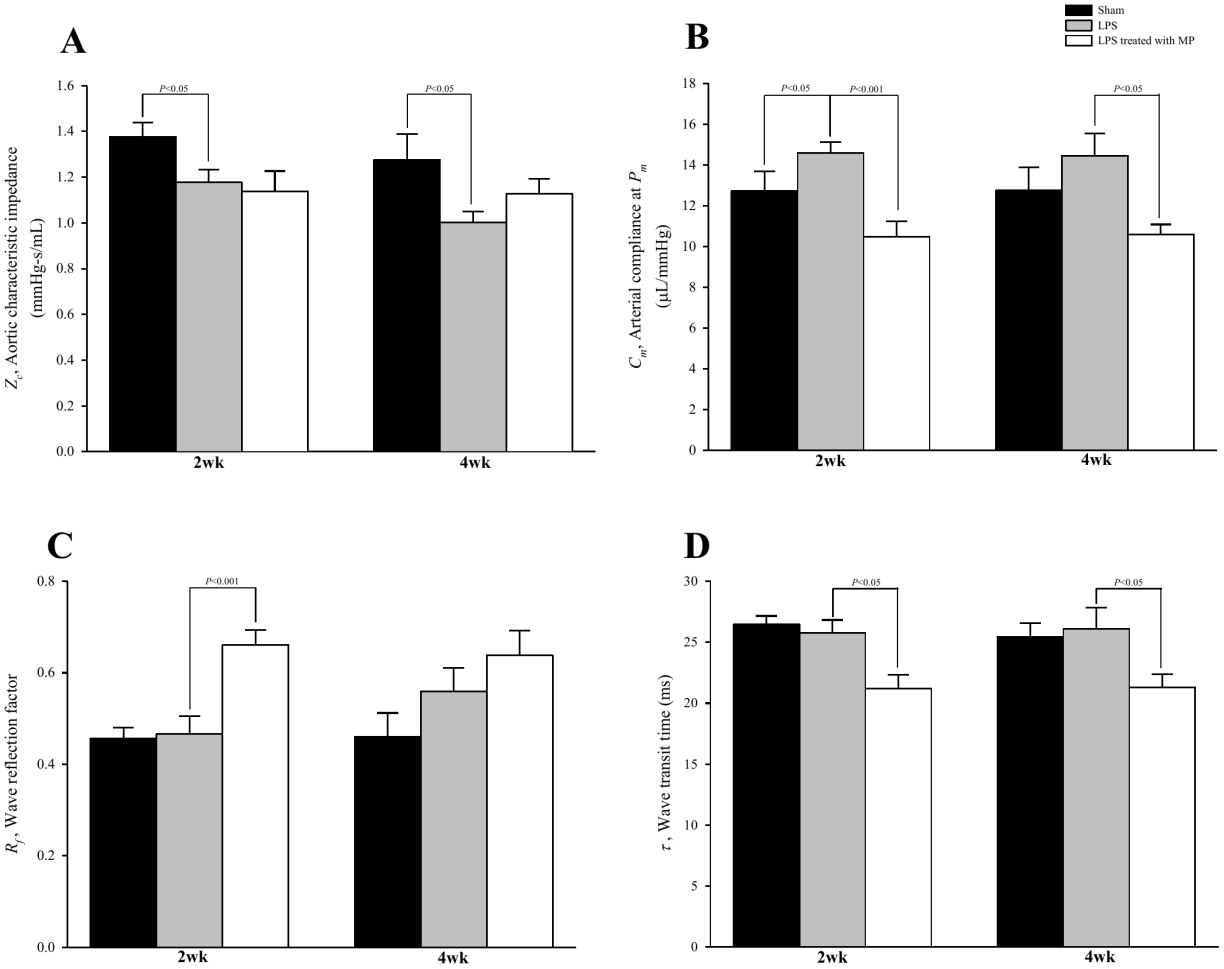
Effects of LPS and MP on aortic characteristic impedance (*Z_c_* in A), systemic arterial compliance at mean aortic pressure (*C_m_* in B), wave reflection factor (*R_f_* in C) and wave transit time (*τ* in D). Data are expressed as mean±s.e. LPS, lipopolysaccharide; MP, methylprednisolone. (*n* = 10 in each group).

Figure 6 shows the effects of LPS and MP on aortic characteristic impedance (*Z_c_* in A), aortic compliance (*C_m_* in B), wave reflection factor (*R_f_* in C) and wave transit time (τ in D). For rats treated with MP, no change was observed for the LPS-induced fall in *Z_c_*. By contrast, MP treatment prevented the significant rise in *C_m_* typically associated with LPS. Neither *R_f_* nor τ changed significantly in rats challenged by LPS, but MP therapy produced a significant increase in *R_f_* and decrease in τ in these animals.

## Discussion

The best characterized AGE receptor is RAGE, which is a multiligand member of the immunoglobulin superfamily of cell surface molecules [26]. Recent evidence has indicated that AGEs, through RAGE, may amplify pro-inflammatory mechanisms in atherogenesis and chronic inflammatory disorders, leading to vascular inflammation, fibrosis, and damage [27]. In this study, we found that long-term LPS challenge resulted in concomitant enhancement of AGEs, RAGE, and iNOS expressions within the vessel wall. The RAGE-AGE interaction associated with LPS stimulation maintained and even amplified inflammatory activities, as manifested by high and prolonged release in CRP, IL-6, and NO plasma levels.

Bucala *et al.* [28] demonstrate that AGEs inactivate NO activity via a rapid chemical reaction. Excessive production of NO is an important player during hypotension in endotoxin-challenged animals [29]. The adverse effects of NO might also, in part, be related to interactions between NO and superoxide anions with subsequent production of peroxynitrite, which is a highly toxic compound to damage VSMCs [30]. Thus, the ability of AGEs to quench NO was supposed to upregulate vascular smooth muscle tone under LPS. However, we found that long-term LPS challenge substantially decreased arterial resistance to blood flow in the peripheral circulation (*R_p_* in Fig. 5D). A decline in *R_p_* suggests that the contractile function of VSMCs may be impaired in resistance arteriole. The contractile dysfunction of VSMCs may diminish vascular smooth muscle tone, which will be responsible for an increase in arteriolar caliber. As a result, the peripheral circulation may engender the vasculature to accommodate more blood, developing capillary complications in LPS-challenged rats. Thus, the detrimental roles of NO and peroxynitrite may account for microvessel damage in inflammatory disorders, which was congruent with the findings by Annane et al. [31]. The decreased arteriolar tone due to LPS was attenuated by MP treatment, as manifested by an increase in *R_p_*. In the absence of any significant changes in AGEs content, the decline in NO production due to MP may explain the prevention of LPS-induced vasodilation. The results we obtained were similar to previous findings that MP therapy inhibited iNOS expression and NO production along with subsequent cytotoxic effects [32], [33].

Arterial wave transit time (τ), which is inversely related to pulse wave velocity, can be used to represent the distensibility of aortas: the stiffer the aortic wall, the shorter the wave transit time, and vice versa [13], [14].

AGEs have been implicated in many of the physiochemical abnormalities in extracellular matrix proteins [34]. These abnormalities caused by AGEs in long-lived proteins include increases in cross-linking and decreases in chemical and proteolytic digestibility. Hence, the pathologic cross-links of aortic collagen by AGEs were supposed to increase aorta stiffness in long-term LPS-challenged rats. However, we found that LPS exerted no influence on the elastic properties of aortas, as shown by no alteration in τ (Fig. 6D). The ability of NO to cause vascular relaxation may counterbalance the AGE-induced arterial stiffening so that the aortic distensibility remained unaltered under LPS. Intriguingly, treating LPS-challenged rats with MP showed an increase in aorta stiffness, as evidenced by the reduction in τ. The MP-induced decline in aortic distensibility paralleled a reduction in NO plasma levels, in the absence of any significant changes in AGEs content. Thus, the diminished vascular relaxation by MP may be responsible for the decreased aortic distensibility as MP did not influence the AGEs-induced arterial stiffening under LPS. From these results, we suggest that for vascular dynamics NO activity may be dominant as a counteraction of AGEs when MP was applied to LPS-induced chronic inflammation in rats.

Changes in timing and magnitude of the pulse wave reflection are considered important determinants of the cardiac muscle cells to adapt to hypertrophy [14]. In this study, LPS challenge did not modify the LV systolic load, which was characterized by no alterations in *R_f_* (Fig. 6C) and τ (Fig. 6D). However, treating LPS-challenged rats with MP contributed to a significant decrease in τ and an increase in *R_f_*, deteriorating the systolic loading conditions for the left ventricle coupled to the arterial system. The enhanced systolic load imposed on the heart by MP was supposed to cause the cardiac muscle cells to hypertrophy. However, the ratio of LV weight to body weight remained unaltered when LPS-challenged rats were treated with MP (Table 1).

We also found that LPS administration produced an increase in lumen diameter of the aortas and caused volume expansion in the vasculature. For large arteries, the square of the lumen radius of the tube is directly related to the pulse wave velocity and is inversely related to the *Z_c_* [13], [14]. In the absence of any significant change in τ, a decline in *Z_c_* (Fig. 6A) implied an increase in aortic diameter under LPS. The LPS-induced increase in aortic diameter was not attenuated by MP treatment; MP produced a decline in τ associated with no alteration in *Z_c_*. In the absence of any significant change in τ, an increase in *C_m_* (Fig. 6B) implied that volume expansion occurred in the rat vasculature under LPS, due to the definition that compliance is equal to distensibility times blood volume [35]. Treating the LPS-challenged rats with MP did not attenuate the volume expansion as MP declined both τ and *C_m_*.

Certain limitations of the current study deserve consideration. Because aortic input impedance cannot be measured in conscious animals, evaluation of the effects of pentobarbital anesthesia on rats is impossible. The results reported here pertain only to measurements made in anesthetized rats in the open-chest condition. This condition might induce changes in aortic pressure profiles and introduce reflex effects not found in the closed-chest condition. The degree to which anesthesia and thoracotomy influence pulsatile hemodynamics in rats is uncertain. However, studies with other animal models suggest that the effects are small relative to the biological and experimental variability between animals [36].

Taken together, long-term LPS challenge resulted in concomitant enhancement of AGEs, RAGE, and iNOS expressions within the vascular wall. The excessive production of NO via iNOS may prevail over the ability of AGEs to quench NO, leading to peripheral vasodilation. By contrast, LPS challenge did not influence the elastic properties of aortas. The counterbalancing effects of NO-mediated vascular relaxation on AGE-induced arterial stiffening may be responsible for the unaltered aortic distensibility. Although treating with MP prevented LPS-induced peripheral vasodilation, MP therapy produced an increase in aorta stiffness. The diminished aortic distensibility by MP paralleled a fall in NO levels, in the absence of any significant changes in AGEs content. From these results, we suggest that MP stiffens aortas and elastic arteries in LPS-induced chronic inflammation in rats, for NO activity may be dominant as a counteraction of AGEs.
